# Research on Measurement of Symbiosis Degree Between National Fitness and the Sports Industry from the Perspective of Collaborative Development

**DOI:** 10.3390/ijerph16122191

**Published:** 2019-06-21

**Authors:** Qing Lan, Chunxiang Liu, Shuang Ling

**Affiliations:** 1 School of Public Administration, Central South University, Changsha 410083, China; jxlanqing@csu.edu.cn (Q.L.); lcx@csu.edu.cn (C.L.); 2 College of Physical Education, Yichun University, Yichun 336000, China

**Keywords:** national fitness, sports industry, symbiotic relationship, collaborative development

## Abstract

Clarifying the symbiotic relationship between national fitness and the sports industry has important theoretical and practical significance for the collaborative development of the two. In order to measure and verify the symbiotic relationship between national fitness and the sports industry, we introduce a “symbiosis degree” model test method for the measurement of the symbiotic relationship during 2000–2017 between the two. Our results show that national fitness and the sports industry have symbiotic sufficient and necessary conditions, for both of which there is a symbiotic relationship, and a non-symmetric reciprocal symbiotic mode long-term. Overall, the impact of national fitness on the sports industry is greater than the impact of the sports industry on national fitness. From a structural perspective, there is a certain difference in the mutual influence of national fitness and the sports industry in the field of sports goods and sports services. In a dynamic forecast, we found that national fitness and the sports industry have the development trend of a symmetric reciprocal symbiotic state. Through theoretical studies, data measured and simulated projections, we believe that the symbiosis degree of the measurement model for the detection and prediction of the symbiotic unit of the symbiotic relationship between them to be of practical value.

## 1. Introduction

The excessive scale [1], the content of demand and the diversity of levels of Chinese participants in national fitness determine that it will become a multi-industry in the form of fitness consumption, media, finance, Internet and so on. Under the background of improving the whole nation’s physical fitness into the national strategy, we should vigorously carry forward the industrial value of national fitness and realize the effective integration and coordinated development of national fitness and the sports industry, so as to promote its social and economic value [2]. Previous studies have shown that there is a high degree of interaction and relevance between national fitness and the sports industry [3,4,5,6], and the two have the basis for collaborative development. However, there are still some problems in the collaborative development of the two, mainly in two aspects. One is the lack of empirical judgment of the degree of interaction between the two, and the second is the lack of dynamic detection of the collaborative development between the two. These problems are likely to lead to the policy imbalance of “taking care of this and losing one.” For example, a scholar has proposed that China’s current economic development level is suitable for the development model of the sports industry with “national fitness as the center” [7]. From the perspective of policy formulation, this is undoubtedly a very valuable research result, but the development model of the sports industry centered on “national fitness as the center” follows the economic market law, whether this is conducive to the coordinated development of the two, especially the development of the sports industry has yet to be further verified. As a result, if we do not clarify these issues, it is easy to embark on a development model of “pure indicator” [8] that pursues the economic aggregate and neglects the law of coordinated development of the two, which could be detrimental to the long-term and healthy development of the two. Therefore, solving these problems is of great significance for studying the collaborative development of national fitness and the sports industry.

In recent years, the application of symbiosis and its theory in sociology and economics has provided a new and creative research path for exploring the collaborative development of national fitness and the sports industry. The essence of collaboration is “cooperation” [9,10], which is consistent with the essence of symbiosis. The theory of symbiosis is to explore the symbiosis between system elements from the perspective of the combination of macro and micro. The essence is “cooperation” and “development” [11,12]. Therefore, from the perspective of the essence, we believe that the collaborative development of national fitness and the sports industry is essentially the symbiotic development of the two. Its core is the symbiotic relationship constituted by the combination of a symbiosis behavior model and symbiosis organization degree among subjects [13]. The purpose of this study is to use the symbiosis theory to examine the symbiotic relationship between national fitness and the sports industry and to explore the theoretical and empirical basis for the coordinated development of the two by trying to answer the two questions raised above.

The previous literature from multiple perspectives on the study of the symbiotic relationship between national fitness and the sports industry provides a theoretical basis, including human capital theory, industrial development theory, and social development theory [14,15,16]. The first two theories analyze the interaction between national fitness and the sports industry from the perspective of economics. Human capital theory believes that human capital plays a decisive role in the economic growth and development of a country [17,18], and that workers’ healthy investment in nutrition, exercise and health care is a healthy capital [19], and the health expenditure of workers is a kind of consumption expenditure [20]. With the growth of sports consumption, it will certainly promote the prosperity of the sports market and accelerate the development of the sports industry. The industrial development theory believes that the sustainable development of industry requires common, coordinated, fair, efficient and multi-dimensional development and the realization of the balance between “need” and “restrictions on need.” However, national fitness and the sports industry cannot only realize the forward coupling development, which reflects the promotion of the sports industry to the development of national fitness but also achieve backward coupling development, reflecting the driving effect of national fitness on the sports industry [21]. These two theories reveal the internal mechanism of the interaction between national fitness and the sports industry and provide theoretical support for the analysis of the existence of a symbiotic relationship between the two, especially the analysis of the symbiotic interface between the two. The social development theory believes that social development is a multidimensional process, including the restructuring of the entire economic and social system [22]. Improving the quality of the people (including physical fitness) is an inherent requirement of social development. The improvement of national physique is inseparable from the demand for sports consumption. However, to meet the sports consumption needs of the Chinese people, it is necessary to provide people with matching sports services according to the new characteristics and changes of different stages of economic and social development. Therefore, in terms of the development of national fitness and the sports industry, the progress of society requires the simultaneous development of the two.

The above theory provides multi-faceted theoretical support for studying the symbiotic relationship between national fitness and the sports industry, but the viewpoint is scattered and requires theoretical integration. Therefore, based on fully absorbing and borrowing these theories, scholars who have tried to apply the symbiosis theory to the collaborative development of national fitness and the sports industry began to theoretically construct a symbiotic system between the two. In terms of the basic elements that constitute the symbiotic relationship between the two, Li (2017) and Lei (2017) both pointed out that national fitness and the sports industry have three basic elements of symbiosis, namely, symbiotic unit, symbiotic mode, and symbiotic environment. National fitness and the sports industry, as the supply and demand side of the sports consumption market, constitutes two symbiotic units of symbiotic relationship. The two have a symbiotic organization model and a symbiotic behavior model. The policy and regulation system, especially the market rule of law environment, is an important part of the symbiotic environment of the two [23,24]. The symbiotic interface is the basic condition that must be met to form a symbiotic relationship. Qian (2016) [21] pointed out that national fitness and the sports industry is a symbiotic and win-win ecosystem, and the “sports consumer market” is its symbiotic interface. Lei (2017) [24] also believes that national fitness and the sports industry are two closely related symbiotic units, and the two have formed a supply-demand relationship with the “sports consumer market” as a symbiotic interface. For the symbiotic state of the two, Zhu (2010) [25] believes that national fitness and the sports industry are in a mutually beneficial, mutually reinforcing, mutually constrained, nested win-win, resonant state. Li (2017) [23] believes that national fitness and the sports industry rely on each other and grow together, which belongs to the continuous symbiosis model.

There are still some deficiencies in the current research. Scholars explored the symbiotic relationship between the two in terms of theory and mechanism. However, most of the previous studies are phenomena analyses, and the method of theoretical interpretation was adopted. The method application is mostly qualitative research, and empirical research is relatively rare, which leads to the weaker explanatory power of the research conclusion. For example, Li (2017) pointed out that the symbiotic relationship and the imperfect institutional mechanism restrict the healthy development of the symbiotic relationship between national fitness and the sports industry. At this stage, national fitness and the sports industry in China belongs to the asymmetric reciprocal symbiosis model [23]. Wang (2017) believes that due to the differences in functions between national fitness and the sports industry and the differences in resources and factor endowments, they tend to be asynchronous, uncoordinated and discordant in development, leading to less interaction between the two in the positive development process [26]. This leads us to believe that the symbiotic relationship between the two is asymmetrical and in a state of partiality. However, the degree of asymmetric symbiosis between the two requires further measurement and identification. In addition, Wang L.Q. (2011) proposed that “Only by accurately grasping the best conjunction point of their benign interaction can realize the win-win and sustainable development of national fitness and the sports industry.” [4], and Qian (2016) argued that “The transformation of the sports industry and the promotion of national fitness organic coupling and docking to form the joint force of the two.” [21], which led us to believe there is a something like a “tipping point” that merges the two, but how to test and accurately determine the “tipping point” between the two needs further theoretical and empirical research. Moreover, the compatibility of qualitative parameters between symbiotic units is a necessary condition for judging the formation of symbiotic relationship. The existing research lacks in-depth research at this point, and further theoretical and empirical tests are needed.

To sum up, scholars have carried out fruitful research on the symbiotic relationship between national fitness and the sports industry from various perspectives, which laid a good theoretical foundation for this research. Therefore, based on absorbing and learning from existing research results, the purpose of this study is to further analyze and test the symbiotic relationship between the two using symbiosis theory. We aim to do so by measuring the symbiosis degree of the two, analyzing the symbiotic linkage relationship between the two, exploring the symbiotic path and mode of the two in sport goods, sport services and other industries, and predicting the dynamic development state of symbiosis between them. In order to achieve this goal, firstly, we need to re-examine the symbiotic conditions of national fitness and the sports industry based on the symbiosis theory. Secondly, establish a symbiosis degree model between national fitness and the sports industry to determine the symbiosis degree and symbiosis coefficient, and verify the symbiotic relationship between the two. Finally, the symbiosis degree measurement model is used to predict the symbiosis trend of national fitness and the sports industry from a dynamic perspective.

## 2. Methodology

Literature analysis served to obtain research data from statistical yearbooks, statistical bulletins, and research reports. Symbiosis degree among variables was measured by the symbiosis measurement model.

### 2.1. Data Sources

According to *the Statistical Bulletin of China’s Sports and related Industries* published by the State Sports General Administration and *the report on the Development of China’s Sports Industry*, we obtained data on the Total Output of National Sports and Related Industries, the Total Output of Manufacturing Industry of Sport Goods and Related Products and the Total Output of Sport Services during the period 2000–2017, and the Total Output of National Sports and Related Industries data in 2008 is from the National Bureau of Statistics (NBS) *the second National Economic Census of the main data Bulletin* [27]. Based on *the Survey report on the current situation of National Mass Sports* published by the State Sports General Administration in 2000, 2007 and 2014, we adopted the method of calculating the Total Sports Consumption of Urban and Rural Residents in *the 2010 China Leisure Development report* [28] published by the Chinese Academy of Social Sciences. We calculated the Total Sport Consumption of Urban and Rural Residents in 2000–2017 and the Consumption of Sport Goods and Sport Services in 2000, 2007 and 2014. The data used to estimate the average annual growth rate of the Per Capita Disposable Income of Urban Residents was all derived from *the Statistical Yearbook* of the National Bureau of Statistics.

### 2.2. Measurement Method

The SPSS 19.0 software (IBM Corp, Armonk, NY, USA) was utilized to analyze the data, to determine the functional relationship between the selected variables, to construct a regression model, and to import the symbiosis measurement model to complete the measurement of the symbiosis degree between variables.

#### 2.2.1. Model for Measurement of Symbiosis

Symbiosis degree is the correlation degree of qualitative parameter changes between two symbiotic units or symbiotic systems. It means that the change rate of the corresponding symbiotic unit quality parameter is caused by that of the symbiotic one [29]. Supposing that in the symbiosis system of national fitness and the sports industry, the main quality parameter of the symbiosis unit is the Total Sports Consumption of Urban and Rural Residents (x), and the main quality parameter of the symbiotic unit sports industry is the Total Output of National Sports Industry and Related Industry (y). The symbiosis degree of national fitness to the sports industry ($\delta_{XY}$) and the symbiosis of the sports industry to national fitness ($\delta_{XY}$) is, respectively, as follows:

(1) $$\delta_{XY}=\frac{dy/y}{dx/x}=\frac{x}{y}\cdot \frac{dy}{dx},$$

(2) $$\delta_{YX}=\frac{dx/x}{dy/y}=\frac{y}{x}\cdot \frac{dx}{dy}.$$

In the formula, $\delta_{XY}$ reflects that the change rate of the highest quality parameter of the sports industry is caused by that of the main quality parameter (x) of national fitness. $\delta_{YX}$ reflects the change rate of the main quality parameter (y), which is caused by the change rate of the main quality parameter of the sports industry, which reflects the promotion effect of the sports industry on national fitness.

Based on determining symbiosis degree, the interaction degree of the quality parameter of the symbiosis unit can be further reflected by the symbiosis coefficient. Supposing the symbiosis coefficient of the quality parameter of national fitness and the sports industry as $\theta_{X}$ and $\theta_{Y}$ respectively, then the symbiosis coefficient $\theta_{X}$ of national fitness to the sports industry and the symbiosis coefficient $\theta_{X}$ of the sports industry to national fitness are expressed as follows:

(3) $$\theta_{X}=\frac{\left|\delta_{XY}\right|}{\left|\delta_{XY}\right|+\left|\delta_{YX}\right|},$$

(4) $$\theta_{Y}=\frac{\left|\delta_{YX}\right|}{\left|\delta_{YX}\right|+\left|\delta_{XY}\right|}.$$

#### 2.2.2. Evaluation of Measurement Results

If $\delta_{XY}=\delta_{YX}>0$, then, $\theta_{X}=\theta_{Y}$, showing that the impact of national fitness on the sports industry is the same as that of the sports industry on national fitness, and the two are in a positive symbiotic state of symmetry and reciprocity; if $\delta_{XY}=\delta_{YX}<0$, national fitness and the sports industry is in the state of opposite symmetry and reciprocal symbiosis; if $\delta_{XY}\neq \delta_{YX}>0$, and $\theta_{X}>\theta_{Y}$, the impact of national fitness on the sports industry is greater than that of the sports industry on national fitness, and the two are in a positive asymmetrical and reciprocal symbiosis state. If $\delta_{XY}\neq \delta_{YX}<0$, and $\theta_{X}>\theta_{Y}$, the impact of national fitness on the sports industry is greater than that of the sports industry, and the two are in the opposite asymmetrical and reciprocal symbiosis. If $\delta_{XY}=0$, $\delta_{YX}>0$, then $\theta_{X}=0$, $\theta_{Y}=1$, and national fitness has no effect on the sports industry, only the sports industry has some effect on national fitness, and both are in the positive side of the symbiosis state; if $\delta_{XY}=0$, $\delta_{YX}<0$, only the sports industry has an effect on national fitness, the two are in reverse and partial symbiosis state; if $\delta_{XY}\times \delta_{YX}<0$, they are in parasitic state, the symbiosis unit whose symbiosis degree is less than zero is parasitic in the symbiosis unit whose symbiosis degree is greater than zero; if $\delta_{XY}=\delta_{YX}=0$, it means that the two have no influence on each other, they are in a concomitant state.

## 3. Theoretical Hypothesis

The word “symbiosis” is from Greek. The concept of symbiosis was first proposed by German mycologist (Anton de Bary) in 1879. He defined symbiosis as a physical connection between one or more members of different species living together. Developed and perfected by Famintsim and Prototaxis, it refers to different species living together in a material connection, forming the relationship between co-existence and co-evolution inhibition [11,23,30,31]. Scott is determined to find the physical relationship between the two sides and considers that symbiosis is an eternal feature of the life cycle of organisms. He defines symbiosis as a state in which two or more features biologically need to balance each other. In most modern biological work, symbiosis is considered to be a living nutritive association of reciprocity [32,33]. The most typical symbiosis is the familiar symbiosis of legume and rhizobium [34]. In the study of insects and birds, Edward O. Wilson of Harvard University made a scientific summary of the symbiosis phenomenon of the population. He thinks that there are three kinds of symbiosis in natural organisms, namely, community parasitism, community preference symbiosis, and community mutualism [35]. Caullery (1952) and Lewis (1973) clearly defined the concepts of symbiosis, reciprocal symbiosis, co-habitation, parasitism and other relationships between organisms of different species [36,37]. Since the middle of this century, the symbiosis method has been applied in the social field. Some sociologists put forward a theory of “symbiosis method” to design the social production system, emphasizing the role and relationship of various factors in the social production system [31]. These studies are of fundamental significance to the establishment of symbiosis theory in the sense of social sciences. Dr. Yuan Chunqing, a Chinese scholar, extended the biology of symbiosis to the social sciences in 1998. He pointed out that “symbiosis” is the relationship between symbiotic units formed according to a certain symbiotic model in a definite symbiotic environment, and the essence of symbiosis is collaboration and cooperation. Symbiosis is not only a social phenomenon but also a social science method. Three analytical frameworks of symbiosis theory are put forward: symbiosis degree analysis, symbiosis interface analysis and symbiosis model analysis [38]. Symbiosis degree is the correlation degree of qualitative parameter change between two symbiotic units or symbiosis systems, which reflects the intrinsic relationship between symbiotic units. Symbiosis degree is the most basic characteristic of the symbiotic system, which reflects the essence and development law of symbiotic system most directly. Symbiosis degree is an important basis for judging the existence of a symbiotic relationship between units and the symbiosis mode between symbiotic units, which provides an important research basis and thought frame for the measurement of symbiosis degree and the judgment of symbiotic relationship between national fitness and the sports industry.

The implementation of the National Fitness Program (NFP) has made people pay more attention to seeking scientific fitness methods suitable for their physical condition and professional characteristics while paying attention to their health, so fitness skills training, counseling, physical fitness testing, health assessment, physical rehabilitation, and so on, have begun to appear as a certain market demand [39]. National fitness consumption demand is a part of the modern healthy life consumption demand. From the economic point of view, it can be understood as the number of sports products that consumers are willing and able to consume in order to meet the needs of a healthy life, under the price level of various market products/services and the time cost [40]. National fitness consumption products can be divided into physical and non-physical forms. The physical form of consumption mainly refers to the purchase of sports clothing, training equipment and other sporting goods of the material-type consumption, and the non-physical form of consumption mainly means to participate in sports activities, fitness training, watching competition performances, sports health care and other service-oriented consumption.

**Figure 1:**
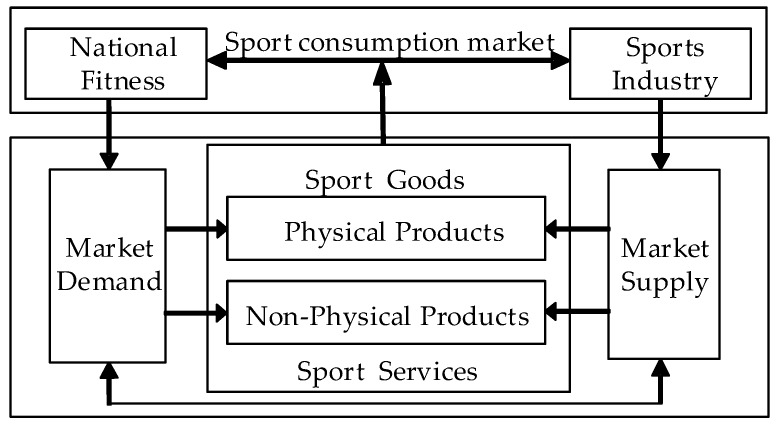
The symbiosis system of national fitness and the sports industry.

The development of the sports industry cannot be separated from sport consumption [41]. Market products provided by the Chinese sports industry are supplied to the residents and the government in the form of final consumption products. The sports industry is a typical consumption industry at present. According to the changing trend of social production structure, the industrial structure (product structure) is determined by the consumption structure [42]. Therefore, in accordance with the national fitness consumption structure, the market products provided by the sports industry are divided into physical and non-physical types. The physical products are all kinds of sports facilities, sports clothing, shoes and hats, training equipment and other sport goods which are provided to society in the form of manufacture. Non-physical products are non-physical forms of living labor to provide various sport services to society to meet people’s physical fitness, entertainment and spiritual need of the product. In the fitness consumption market, the two symbiotic units of national fitness and the sports industry form a supply-demand relationship, as shown in Figure 1.

The analysis shows that national fitness and the sports industry are two relatively independent subsystems, and they are in the same sport consumption market system and have a symbiotic relationship, which can be used to measure the symbiosis degree. The measurement of symbiosis degree can be used to further analyze the synergetic effect and symbiosis between the two systems.

### 3.1. Symbiotic Relationship between National Fitness and the Sports Industry

The existence of symbiosis degree is based on the compatibility of quality parameters, which are the feature of mutual expression of quality parameters of symbiosis units. Compatibility of quality parameters is not only the logical criterion of symbiosis relation but also the symbiosis organization mode of the symbiosis unit. The mathematical expression is that if $Z_{i}=f\left(Z_{j}\right)$, then i and j may form a symbiotic relationship, where $f\left(Z_{j}\right)$ can be a random function, a discontinuous function or a continuous function, and different functions correspond to different symbiotic patterns. If $f\left(Z_{j}\right)$ is a definite continuous function (linear or non-linear), then a continuous symbiosis mode or an integrated symbiosis mode may be formed between i and j. In the symbiosis system of national fitness and the sports industry, the quality parameter between national fitness and the sports industry is the supply and demand for sports commodities, which reflects the internal connection and corresponding relationship between the two. According to the symbiosis theory, the primary quality parameter can reflect the corresponding overall relationship between the two symbiotic units. The Total Sports Consumption of Urban and Rural Residents can reflect the overall demand for sports goods. The Total Output of National Sports Industry and Related Industries can reflect the overall supply of sports goods. Therefore, the two variables correspond to each other and become the primary quality parameters between the two. The transmission of material, energy and information between supply and demand of sport goods in the sports consumption market satisfies $T\in \left[{Τ}_{o},{Τ}_{p}\right]\;{Τ}_{0}\neq {Τ}_{p}$, $\Psi \in \left[\Psi_{0},\Psi_{p}\right]\Psi_{0}\neq \Psi_{p}$ on time Τ and space Ψ, according to symbiosis theory, on the basis of satisfying the above-mentioned space-time relations. If national fitness and the sports industry Symbiosis Degree $\delta_{XY}<0$, $\delta_{YX}>0$, then it may be concluded that national fitness and the sports industry are in the continuous symbiosis relations. This is consistent with the logical judgment of the existing policy, that is, there is an inevitable connection between national fitness and the sports industry, and the changes between them have a positive impact on each other. Such as, “develop the sports industry, promote national fitness,” “give full play to promotion effect national fitness’s on development of the sports industry” [43]. Therefore, the following hypothesis is proposed: 

H1: The quality parameters of national fitness and the sports industry are compatible. National fitness and the sports industry have a symbiotic relationship and are in a continuous symbiosis model.

### 3.2. The Symbiotic State between National Fitness and the Sports Industry

The development of the sports industry is inseparable from the pull of sport consumption. Market products provided by the Chinese sports industry are supplied to the residents and the government in the form for final consumption, and the sports industry is a typical final consumption industry. From the perspective of the trend of changs in the social production structure, the industrial structure (product structure) is determined by the consumption structure [41]. In the symbiosis system of national fitness and the sports industry, national fitness is on the demand side of sport goods, and the sports industry is on the supply side of sport goods. National fitness is the basis of the development of the sports industry. Promoting national fitness and sports consumption is an important guarantee to speed up the development of the sports industry. Vigorous sport consumption demand brought by national fitness directly promotes the development of the sports industry. Since 2000, China’s sport goods market has been increasing at a double-digit rate every year. Such a huge market has brought abundant opportunities for sport goods manufacturers. In 2016, China’s sport goods industry (sportswear, sneakers, sports shoes, sports clothing, sports shoes, the manufacturing and selling of sports equipment and related sport products) reached 307.7 billion yuan, with a growth rate of 11.65% [44]. According to the NFP (2016–2020), by 2020, 40% of the population will actively participate in all kinds of sports activities, and the total size of sports consumption will reach 1.5 trillion yuan. National fitness has become the power source to promote the advancement of the sports industry. According to the principle of compatibility of qualitative parameters, if there is an asymmetrical relationship between the two, it will be reflected in the primary parameters. The symbiotic state of the two can be further judged by measuring the symbiosis degree of the primary quality parameters. Therefore, the following hypothesis is put forward: 

H2: National fitness and the sports industry are in an asymmetric and reciprocal symbiosis, and the impact of national fitness on the sports industry is greater than that of the sports industry on national fitness.

### 3.3. Symbiotic Model between National Fitness and the Sports Industry Structure

With the development of economy and the improvement of the residents’ income level, people’s sport consumption concept, consumption consciousness and consumption structure are also changing constantly, resulting in the adjustment of the sports industry structure. Therefore, in a certain period, the relationship among the various elements that constitute national fitness and sports industrial structure is sure to experience a certain fluctuation. According to the theory of Industrial Economics, the development of the industrialized economy extends from the field of material production to that of immaterial production. The material production in the sports industry is mainly in the sport equipment industry. According to the consumption theory, the consumer’s consumption is the motive of satisfying demand, and human demand primarily realizes the material demand and then the non-material demand. Therefore, consumer consumption of sport goods also follows the demand process from material products to non-material products [44]. In 2015, figures from the State Sports General Administration and the National Bureau of Statistics of China showed that the manufacturing of sporting goods and related products accounted for 65.7% of the country’s total output in the sports industry; and the sport services sector accounted for 33.4% of the country’s total output. According to the survey of the State Sports General Administration in 2014, among sport consumers, the highest percentage of people buying sportswear was 93.9%, and the percentage of people buying various sport services was under 10%. The sports industry structure and the sport consumption structure show an obvious non-symmetry development condition, and the material exchange of national fitness and the sports industry in the field of sport equipment is obviously more than that in the field of sport services. The supply and demand exchange of the two in the sport goods field is expressed through the Consumption of Sport Goods and the Total Output of Sport Goods and Related Products Manufacturing, and in the sport services field is expressed through the Consumption of Sport Services and the Total Output of Sport Services. The model of symbiosis between the internal structure of the two can be determined by the measuring the symbiosis degree of the two groups of quality parameters. Therefore, the following hypothesis is put forward: 

H3: National fitness and the sports industry are in the mode of asymmetric mutualism, and the compatibility degree of quality parameters of sport goods is higher than that of sport services.

### 3.4. Evolutionary Trend of Symbiosis System between National Fitness and the Sports Industry

The symbiosis principle reveals that symbiosis is the consistent direction of symbiosis system evolution and the fundamental law of evolution in biology and human society. It is the inevitable trend of the symbiosis between nature and human society [7]. The relationship between national fitness and the sports industry is interrelated and cooperatively developed. On one hand, national fitness is the basis and motivating power of the sustainable development of the sports industry. It has created a good market environment for the sports industry, and it has brought about the sport consumption and the structural changes in the consumption [6]. On the other hand, the healthy development of the sports industry is the condition and guarantee for all people to keep fit, thus forming a social sport services system suitable for the socialist market economy. The new policy of the sports industry enriches the connotation of the national fitness policy system, especially as the publication of the State Council document No. 46 provides additional opportunities for its development and leads national fitness to a higher level [45]. With the favorable conditions of institute, policy and market environment, national fitness and the sports industry have a good trend of symmetrical symbiosis. Further analysis of the symbiosis degree of the primary quality parameters can be used to judge the symbiotic development trend of the two. Therefore, the following hypothesis is put forward: 

H4: National fitness and the sports industry are improved toward symmetrical and mutually beneficial symbiosis.

## 4. Empirical Test and Result Analysis

### 4.1. Symbiotic Relationship between National Fitness and the Sports Industry

The Total Sports Consumption of Urban and Rural Residents (TSCURR) and the Total Output of National Sports Industry and Related Industries (TONSIRI) were selected as the primary quality parameters of national fitness and the sports industry, respectively. Table 1 shows the data of primary qualitative parameters from 2000 to 2017.

**Figure 2:**
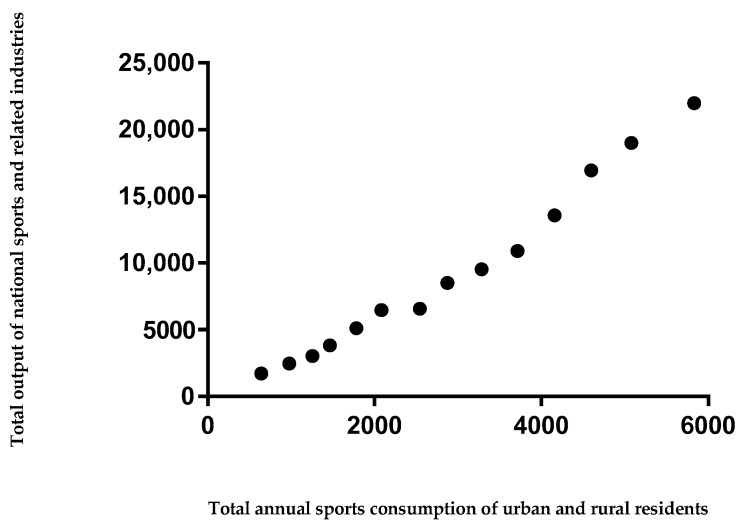
Scatter plot of the primary quality parameters.

SPSS software was used to test the single sample K−S normality of two variables. The results showed that the *p* values of x and y were 0.991 and 0.855, respectively, both of which are greater than 0.1, which indicated that the samples had a normal distribution as a whole. By creating a scatterplot of two variables, we can see that all the points are close to one line, and there is a significant linear relationship between the two variables, which shows that the functional relationship between the two variables is suitable to be represented by a univariate linear model (Figure 2)

The results of the univariate linear regression analysis of two variables are as follows:

(5) x=578.078+0.248y,

(6) y=−2052.374+3.932x.

The correlation coefficient of the two models is R=0.988, and the determining coefficient is $R^{2}=0.976$, which indicates that the fitness of the model is high. The results of model ANOVA: $F=491.712>F_{0.05}\left(1,\;12\right)=4.75$, show that the linear relationship of the model is significant at the 95% confidence level.

According to models (1) and (2), Equations (5) and (6), the following results are obtained:

(7) $$\delta_{XY}=3.932\cdot \frac{x}{y}=\frac{3.932x}{-2052.374+3.932x},$$

(8) $$\delta_{YX}=0.248\cdot \frac{y}{x}=\frac{0.248y}{578.078+0.248y}.$$

From models (7) and (8), the symbiosis degree of between the two in 2000–2017 can be obtained. The resulting symbiosis degree values were introduced into equations (3) and (4) to get the co-occurrence coefficient of national fitness and the sports industry (Table 2).

The results of data analysis show that the primary quality parameter of national fitness and the sports industry is the Total Sports Consumption of Urban and Rural Residents x and the Total Output of National Sports Industry and Related Industries y, with a function relationship, that is $Z_{x}=f\left(Z_{y}\right)$, $Z_{y}=f\left(Z_{x}\right)$, and $f\left(Z_{x}\right)$, $f\left(Z_{y}\right)$ is a continuous function. According to the principle of compatibility of quality parameters, it can be concluded that the quality parameters of national fitness and the sports industry have the characteristics of mutual expression and inherent interrelation. From 2000 to 2017, the symbiosis degree of national fitness and the sports industry is $\delta_{XY}>0$, $\delta_{YX}>0$. According to the principle of quality parameter compatibility, if $Z_{i}=f\left(Z_{j}\right)$ is a definite interval continuous function, then it is easy to form a constant symbiosis model between them, which can be further judged to form a constant symbiosis relationship between the two. Hypothesis H1 is valid.

### 4.2. Symbiotic State between National Fitness and the Sports Industry

Table 2 shows that the symbiosis degree of national fitness and the sports industry $\delta_{XY}\neq \delta_{YX}>0$ in 2000–2017, both are in a positive asymmetrical and reciprocal symbiosis state. The symbiosis coefficient $\theta_{X}$ of national fitness to the sports industry is more greater than $\theta_{Y}$ of the sports industry to national fitness. In the symbiosis system of national fitness and the sports industry, the influence of national fitness on the sports industry is higher than that of the sports industry on national fitness. Hypothesis H2 is valid.

### 4.3. Symbiotic Model between National Fitness and the Sports Industry Structure

Two groups of supply and demand parameters were selected to analyze the symbiosis degree and relationship. Table 3 is the two groups of quality parameter data corresponding to national fitness and the sports industry. Similarly, the regression analysis of two groups of variables: the Consumption of Sport Goods $x_{1}$, the Total Output of Sport Goods and Related Products Manufacturing $y_{1}$, the Consumption of Sport Services $x_{2}$ and the Total Output of Sport Services $y_{2}$, were made, respectively. The symbiosis degree and symbiosis relationship of the two groups of variables were derived from the symbiosis measurement formula. The results of data analysis are shown in Table 4.

The results of data analysis show that in the field of sport goods, symbiosis degree $\delta_{x_{1}y_{1}}\neq \delta_{y_{1}x_{1}}>0$ was in asymmetrical positive symbiosis from 2000 to 2014 in national fitness and the sports industry. In the field of sport services, from 2000 to 2014, national fitness and the sports industry experienced a transformative process from parasitic relationship to reciprocal symbiosis relationship, which is in line with the development reality of the transformation and upgrading of the sports industrial structure from the physical type to the service type in China. In the field of sport goods, from 2000 to 2014, the impact of national fitness on the sports industry and the promotion gradually tended to be symmetry and reciprocal symbiosis. In the field of sport services, the promotion of national fitness to the sports industry is much higher than that of the sports industry. In 2014, the symbiosis degree of national fitness to the sports industry was 1.555. Symbiosis degree of the sports industry to national fitness is much higher than that of the sports industry, which indicates that there is still a lot of room for the transformation and upgrading of the sports industry structure in China.

According to the symbiosis theory, for a positive symbiosis, the higher the symbiosis degree, the higher the marginal density energy of symbiosis system, which indicates that the closer the relationship between symbiotic units is, the higher the compatibility degree of qualitative parameters is. Comparing the symbiosis degree of two groups of quality parameters of national fitness and the sports industry, the symbiosis degree of sport services quality parameter in 2000, $\delta_{x_{2}y_{2}}=-7.004$, the sports industry has a one-way material flow to national fitness, and the data of this year cannot be compared. The comparison of the remaining data shows that the symbiosis degree of the sport services quality parameter is higher than that of the sport quality parameter in the aspect of national fitness affecting the sports industry. In the perspective of the influence of the sports industry on national fitness, the symbiosis degree of sport goods parameter is higher than that of the sport services quality parameter. The analysis results do not fully support that the symbiosis degree of sport goods parameters is higher than that of sport services quality parameters. Hypothesis H3 is not quite valid.

### 4.4. Evolutionary Trend of Symbiosis System between National Fitness and the Sports Industry

**Figure 3:**
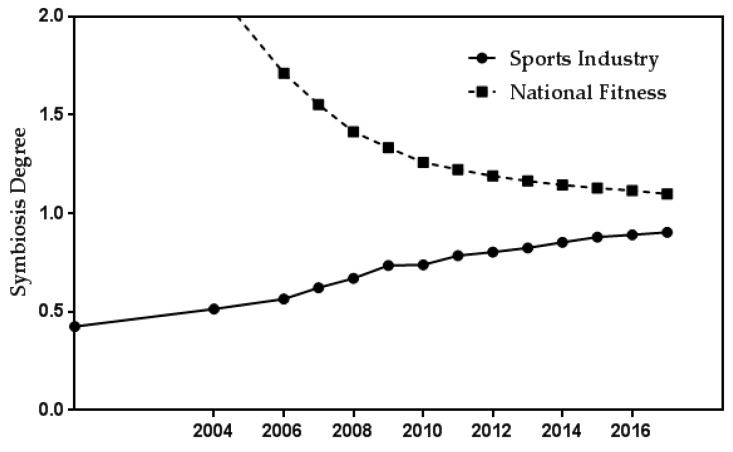
Time series diagram of national fitness and the sports industry symbiosis degree.

To further observe the symbiosis trend of national fitness and the sports industry, the time series diagram of symbiosis degree of national fitness and the sports industry can be made according to the results of Table 2 data analysis (Figure 3). Figure 3 demonstrates that the symbiosis degree of national fitness and the sports industry gradually approaches the value of 1 from 2000 to 2017. When both values are 1, the symbiosis coefficient $\theta_{i}=\theta_{j}=\frac{1}{2}$, and the symbiosis coefficient between the two will reach symmetrical symbiosis. Therefore, the symbiosis system of national fitness and the sports industry has the trend of positive symmetry and reciprocal symbiosis.

To further build the model to predict the symbiosis of national fitness and the sports industry, suppose that time series t(t=1,2,3,⋯,12) means 2006, 2007, 2008, …, 2017. The function of $\delta_{XY}$, $\delta_{YX}$ to T is obtained by using SPSS software, and the results are as follows:

(9) $$In\delta_{XY}=f\left(t\right)=In1.726-0.187In\left(t\right),$$

(10) $$In\delta_{YX}=f\left(t\right)=In0.551+0.197In\left(t\right).$$

In Equation (9), the determining coefficient $R^{2}=0.994$ shows that the regression equation can invert 99.4% of the information of $\delta_{XY}$ with the change of time, and the fitting degree of the model is very high. The result of ANOVA: $F=1537.98>F_{0.05}(1,10)=4.96$ shows that the linear relationship of the model is significant at the 95% confidence level. In formula (10), the determinative coefficient $R^{2}=0.990$, which indicates that the regression equation can reflect 99% of the information of $\delta_{YX}$ with the change of time, and the fitting degree of the model is high. The result of ANOVA:$F=958.699>F_{0.05}(1,10)=4.96$ shows that the linear relationship of the model is significant at the 95% confidence level.

Supposing $\delta_{XY}=\delta_{YX}>0$, according to Equations (9) and (10), symmetric reciprocal symbiosis time of national fitness and the sports industry can be obtained as t=19.56≈20. It is anticipated that national fitness and the sports industry will be in a positive symmetry and reciprocity symbiosis state in 2025.

According to Equations (7) and (8), to make national fitness and the sports industry realize the positive symbiosis of symmetry and reciprocity, that is, $\delta_{XY}=\delta_{YX}>0$, then the combined relationship that x and y need to satisfy that shown in Equation (11).

(11) x=0.251y.

Basing on Equation (11), examples of symmetric reciprocal symbiosis between the two are given (Table 5). If national fitness and the sports industry symbiosis degree $\delta_{XY}=\delta_{YX}=0.987$, symbiosis coefficient $\theta_{X}=\theta_{Y}$, then national fitness and the sports industry can achieve a long-term symbiosis of symmetry and reciprocity.

According to Table 5, under the premise of long-term symbiotic and mutually beneficial development of national fitness and the sports industry, if the development goal of the sports industry totaling more than 3 trillion yuan proposed in the 13th Five-Year Plan of the State Sports General Administration is to be realized, the Total Sports Consumption of Urban and Rural Residents (TSCURR) will exceed 753 billion yuan by then, which means that during the 13th Five-Year Plan period, the average annual growth rate of TSCURR needs to be maintained at a relatively high level of about 11%. The 13th Five-Year Plan of China that by 2020, Gross Domestic Product(GDP) and the Per Capita Income of Urban and Rural Residents(PCIURR) will double that of 2010. It can be concluded that during the 13th Five-Year Plan period, the average annual growth rate of PCIURR will reach about 10%, which provides a strong driving force for the growth of TSCURR. With the promotion of national fitness into a national strategy, the vigorous promotion of policy dividends, the strong release of sport consumption market demand, sport consumption will achieve the expected goal, and national fitness and the sports industry will achieve symbiotic and mutually beneficial development.

In conclusion, Hypothesis H4 is valid.

## 5. Discussion

We adopted the symbiosis theory to explore the symbiotic relationship between national fitness and the sports industry. The results imply that national fitness and the sports industry, as two relatively independent subsystems (symbiotic units), are in the social market (symbiotic system). Specifically, the two exchange materials, information and energy (qualitative parameters) are in the sports consumption market (symbiotic interface), and this exchange is affected by the external environment (symbiotic environment) such as market laws, policies and so on. According to the requirements of symbiosis theory for symbiotic elements, we think that there is a symbiosis phenomenon and the basic element conditions of symbiosis between the two, which is consistent with most existing research viewpoints [5,23,24,26]. The key point is that we found the main quality parameters of the two are compatible by building the symbiosis degree measurement model. The compatibility of principal parameters is a necessary condition for the symbiotic relationship between the two. The empirical results verified the viewpoint [21,23,24,25] that there is a symbiotic relationship between national fitness and the sports industry. 

Further, we found that national fitness and the sports industry is in a positive, asymmetrical symbiosis model. This means that the two have a favorable impact on each other, but national fitness has more impact and promotion on the sports industry in the field of sports services. The sports industry has a greater impact on national fitness in the field of sports goods, which is consistent with the views of most existing studies. Most scholars believe that the demand for sports and the supply of sports in China are not balanced. On the supply side, there is still a problem of insufficient effective supply. The sports service industry is still a “short slab” in the development of the sports industry in China, which does not match with the demand of national fitness. This structural imbalance, which focuses on manufacturing and neglects service, needs to be resolved through high-quality development [24,46,47]. One of the important reasons may be that China’s sports industry is a typical “final consumption” industry, its development cannot be separated from the promotion and pull of fitness consumption, and the development concept and target positioning has not yet realized the transformation of “consumer-oriented.” As a result, the sports industry was in a passive position for a long time, especially in the sports service industry. As noticed by Shen (2016), the integration of the sports industry with the outside world is not enough, the quality and supply efficiency of sports services and products are not high, and the supply is insufficient, which makes it difficult to meet the growing demand for sports consumption of the mass [48]. 

Finally, on the issue of the symbiotic development trend of national fitness and the sports industry, the results also verified the viewpoint that “symmetrical reciprocity symbiosis is the same direction of symbiotic system evolution” [41]. National fitness and the sports industry have a dynamic trend of symbiotic development towards symmetry and reciprocity. The prediction results of the model show that national fitness and the sports industry will be close to the ideal state of positive symmetry and reciprocity symbiosis in 2025. We think that the ideal state is only a kind of unfulfilled goal state, and the predicted results are also based on the existing data analysis. In practice, the coordinated development of the two will inevitably be affected and restricted by more symbiotic environmental factors. As Qian (2016) noticed, if there is no scientific theoretical guidance and policy guidance, it will cause the development of national fitness and the sports industry to be misplaced or alienated [21]. In this case, the state should promote the coordinated development of national fitness and the sports industry to the height of national development as soon as possible, and form the coordinated development strategy of the two.

## 6. Conclusions

The purpose of this study was to investigate the symbiotic relationship between national fitness and the sports industry. To achieve this goal, we built a model, and called it a symbiosis degree measurement model, to verify their symbiotic relationship. These research findings indicate that national fitness and the sports industry is a mutually beneficial symbiosis system, and that the coordinated development can also expect to be realized. It means that the government can promote their mutual development by investigating their common interests, which can be applied to some field, such as, “effective utilization of Gymnasium resource” [49], “withdrawing from sports events” [50], “promoting the deep integration of national fitness and overall health” [51] and “promoting the substantial integration of intelligent sports and national fitness” [52]. In addition, this synergetic pattern can improve not only the industrialization of sport events and health services, but also drive the development of national fitness, which can produce the effect of 1+1>2. Besides, the asymmetric symbiotic model of national fitness and the sports industry was also indicated in our model, which emphasized that national fitness has a bigger effect on the sports industry. However, in practice, due to the differences of ideals, target and structure between national fitness and the sports industry, the two show interactive difference in the development process. It means that the government should pay more attention to the supply-side structural reform in the sports industry [53]. Specifically, the government should enhance the role of the sports industry in national fitness, and improve their collaborative development. Finally, a key point, we established a model of the symbiosis regression equation to detect and predict the symbiotic relationship between national fitness and the sports industry from a dynamic perspective, which has a good policy reference value for the formulation of economic development planning. For example, in the process of supply-side reform, we can predict the goals that sports consumption needs to achieve according to the development goals of the sports industry, and carry on the short-term or long-term detection of the speed on both sides of supply and demand. If there is a great deviation, we can timely use various policy tools to adjust, so as to achieve the sustainable and collaborative development of the two.

This study reveals the symbiotic state of national fitness and the sports industry, provides a theoretical and empirical basis to accurately determine the symbiotic relationship between the two, and expands the scope of application of symbiosis theory. It may provide a reference for subsequent research on the empirical method. However, based on the problem of obtaining data, the three groups of qualitative parameters used in this study can only reveal the symbiotic relationship between the two as a whole. Due to the rich and continuous expansion of the sectors contained in national fitness and the sports industry, this study inevitably has some limitations. With the prosperity of national fitness and the continuous improvement of the sports industry classification and national classification statistics, more variables (qualitative parameters) should be supplemented for theoretical testing and model correction in time. For example, the sports service field has been in a passive position for a long time in the symbiotic relationship between national fitness and the sports industry, which was initially verified by this paper. However, with the focus of supply-side reform, the sports service industry needs us to further adopt “scale-up study” in the future. Then, the “fine” monitoring data on both sides of the supply and demand of the sports service consumer market is crucial for future empirical research. In addition, the symbiotic system of national fitness and the sports industry is greatly affected by the external environment, especially the institutional and policy environment. Therefore, these environmental factors should be considered in future research, and analyzing how these factors affect the symbiotic relationship between the two.

## Figures and Tables

**Table 1:** The primary quality parameters data (unit: RMB 100 million).

Year	TSCURR x	TONSIRI y
2000	643	1726
2004	978	2470
2006	1255	3022
2007	1466	3828
2008	1783	5119
2009	2083	6468
2010	2543	6563
2011	2874	8520
2012	3284	9526
2013	3714	10,913
2014	4157	13,575
2015	4599	16,952
2016	5077	19,011
2017	5832	21,988

**Table 2:** Symbiosis degree and model of national fitness (NF) and the sports industry (SI).

Year	$\delta_{XY}$	$\delta_{YX}$	$\theta_{X}$	$\theta_{Y}$	Symbiosis Model Evaluation
2000	5.329	0.425	0.926	0.074	$\delta_{XY}\neq \delta_{YX}>0$, positive asymmetric reciprocal symbiosis $\theta_{X}>\theta_{Y}$, NF has a more significant impact on SI
2004	2.146	0.514	0.807	0.193	$\delta_{XY}\neq \delta_{YX}>0$, positive asymmetric reciprocal symbiosis $\theta_{X}>\theta_{Y}$, NF has a more significant impact on SI
2006	1.712	0.565	0.752	0.248	$\delta_{XY}\neq \delta_{YX}>0$, positive asymmetric reciprocal symbiosis $\theta_{X}>\theta_{Y}$, NF has a more significant impact on SI
2007	1.553	0.622	0.714	0.286	$\delta_{XY}\neq \delta_{YX}>0$, positive asymmetric reciprocal symbiosis $\theta_{X}>\theta_{Y}$, NF has a more significant impact on SI
2008	1.414	0.670	0.678	0.322	$\delta_{XY}\neq \delta_{YX}>0$, positive asymmetric reciprocal symbiosis $\theta_{X}>\theta_{Y}$, NF has a more significant impact on SI
2009	1.334	0.735	0.645	0.355	$\delta_{XY}\neq \delta_{YX}>0$, positive asymmetric reciprocal symbiosis $\theta_{X}>\theta_{Y}$, NF has a more significant impact on SI
2010	1.258	0.738	0.630	0.370	$\delta_{XY}\neq \delta_{YX}>0$, positive asymmetric reciprocal symbiosis $\theta_{X}>\theta_{Y}$, NF has a more significant impact on SI
2011	1.222	0.785	0.609	0.391	$\delta_{XY}\neq \delta_{YX}>0$, positive asymmetric reciprocal symbiosis $\theta_{X}>\theta_{Y}$, NF has a more significant impact on SI
2012	1.189	0.803	0.597	0.403	$\delta_{XY}\neq \delta_{YX}>0$, positive asymmetric reciprocal symbiosis $\theta_{X}>\theta_{Y}$, NF has a more significant impact on SI
2013	1.164	0.824	0.585	0.415	$\delta_{XY}\neq \delta_{YX}>0$, positive asymmetric reciprocal symbiosis $\theta_{X}>\theta_{Y}$, NF has a more significant impact on SI
2014	1.144	0.853	0.573	0.427	$\delta_{XY}\neq \delta_{YX}>0$, positive asymmetric reciprocal symbiosis $\theta_{X}>\theta_{Y}$, NF has a more significant impact on SI
2015	1.128	0.879	0.562	0.438	$\delta_{XY}\neq \delta_{YX}>0$, positive asymmetric reciprocal symbiosis $\theta_{X}>\theta_{Y}$, NF has a more significant impact on SI
2016	1.115	0.891	0.556	0.444	$\delta_{XY}\neq \delta_{YX}>0$, positive asymmetric reciprocal symbiosis $\theta_{X}>\theta_{Y}$, NF has a more significant impact on SI
2017	1.098	0.904	0.548	0.452	$\delta_{XY}\neq \delta_{YX}>0$, positive asymmetric reciprocal symbiosis $\theta_{X}>\theta_{Y}$, NF has a more significant impact on SI

**Table 3:** Two groups of NF and SI quality parameter data (unit: RMB 100 million).

Year	$y_{1},$	$y_{2},$	$x_{1}$	$x_{2}$
2000	407	252	584	199
2007	3188	640	1151	357
2014	8918	4657	3308	637
2015	11,238	5714	3662	706

**Table 4:** Symbiosis degree and model of sport goods and sport services in NF and SI.

Indicators	2000	2007	2014
$\delta_{x_{1}y_{1}}$	2.391	1.419	1.114
$\delta_{y_{1}x_{1}}$	0.251	0.725	0.88
$\theta_{x_{1}}$	0.905	0.662	0.559
$\theta_{y_{1}}$	0.095	0.338	0.441
$\delta_{x_{2}y_{2}}$	−7.004	2.755	1.555
$\delta_{y_{2}x_{2}}$	0.081	0.184	0.621
$\theta_{x_{2}}$	0.989	0.938	0.715
$\theta_{y_{2}}$	0.011	0.062	0.285
Evaluation of the symbiosis Model of Sport Goods	$\delta_{x_{1}y_{1}}\neq \delta_{y_{1}x_{1}}>0$ positive asymmetric reciprocal symbiosis $\theta_{x_{1}}>\theta_{y_{1}}$ NF has a more significant impact on SI	$\delta_{x_{1}y_{1}}\neq \delta_{y_{1}x_{1}}>0$ positive asymmetric reciprocal symbiosis $\theta_{x_{1}}>\theta_{y_{1}}$ NF has a more significant impact on SI	$\delta_{x_{1}y_{1}}\neq \delta_{y_{1}x_{1}}>0$ positive asymmetric reciprocal symbiosis $\theta_{x_{1}}>\theta_{y_{1}}$ NF has a more significant impact on SI
Evaluation of the symbiosis Model of Sport Services	$\delta_{y_{2}x_{2}}>0$, $\delta_{x_{2}y_{2}}<0$ National fitness is in parasitic State	$\delta_{x_{2}y_{2}}\neq \delta_{y_{2}x_{2}}>0$ positive asymmetric reciprocal symbiosis $\theta_{x_{2}}>\theta_{y_{2}}$, NF has a more significant impact on SI	$\delta_{x_{2}y_{2}}\neq \delta_{y_{2}x_{2}}>0$ positive asymmetric reciprocal symbiosis $\theta_{x_{2}}>\theta_{y_{2}}$, NF has a more significant impact on SI

**Table 5:** Examples of symmetric reciprocal symbiosis combined relationship between NF and SI.

Combined Relationship	The Total Sports Consumption of Urban and Rural Residents (unit: RMB 100 million) x	The Total Output of the Sports Industry (unit: RMB 100 million) y	$\delta_{XY}=3.932\cdot \frac{x}{y}$	$\delta_{YX}=0.248\cdot \frac{y}{x}$
1	5020	20,000	0.987	0.987
2	7530	30,000	0.987	0.987
3	10,040	40,000	0.987	0.987
4	12,550	50,000	0.987	0.987
……	x=0.251y		0.987	0.987
